# Novel approach in HIV therapy: lipid nanoparticle-based mRNA delivery technology using LNP X-HIV Tat-CRISPRa system

**DOI:** 10.34172/apb.025.45973

**Published:** 2025-08-31

**Authors:** Aminath Efa Ibrahim, Kannan Subbaram, Razana Faiz, Sheeza Ali

**Affiliations:** School of Medicine, The Maldives National University, Male’, Maldives

## To Editor,

 There are several antiretroviral agents available for the treatment of HIV patients. These drugs vary in efficacy and cannot offer complete elimination of HIV virions from the body. Novel and more efficient anti-HIV therapeutic technologies are being developed in many parts of the world. During infection, HIV undergoes latency as a provirus inside CD4 + cells. Currently available antiretroviral agents cannot reverse the latent HIV attached to the host DNA.^1^ Now, there is a breakthrough in bringing out latent HIV outside the human genome. The use of a latency reversal agent with the mRNA delivery method shows a very promising scope for HIV treatment.^2^ This method uses lipid nanoparticle-based mRNA technology with the LNP X-HIV Tat-CRISPRa system that delivers mRNA into infected CD + cells. The latent HIV genome is transcribed, released from the human genome, and subsequently eliminated by the antiretroviral agents.

 The Joint United Nations Program on HIV/AIDS reports that there are 40 million individuals living with HIV, with 20.8 million cases reported in eastern and southern Africa, which is one of the areas severely struck by the HIV pandemic.^3^ HIV transmission can occur by various ways, such as through sexual contact, blood transfusion, contaminated needles or from mother to infant. During infection, the virus enters CD4 + cells, and viral RNA is converted to DNA by reverse transcription. This viral DNA, called a provirus, gets attached to the host DNA as provirus and undergoes latency. However, the infected cells can only produce virions if they receive an activation signal. With this, the viral mRNA is translated, producing viral peptides that are used to form new viral structures. Once the virus is assembled, it buds from the cell surface, incorporating the host cell’s membrane, which allows it to infect other CD4 + cells. As the virus replicates, there is a high level of viremia detected.^4^

 The course of HIV infection varies throughout eight to ten years, as the disease progresses through the various phases. Patients whose viral replication is not controlled adequately may have a critically low level of CD4 + cell count after several years of initial infection. The Centers for Disease Control and Prevention (CDC) includes all HIV-infected individuals > 5 years of age with a CD4 + cell count below < 200 cells/mm³ within the definition of acquired immunodeficiency syndrome (AIDS).^4^ These individuals are at high risk of developing AIDS-defining illnesses that can be life-threatening. Since the first reported case of AIDS in 1981, the scientific community has approved more than 30 antiretroviral drugs, such as nucleoside reverse transcriptase inhibitors (NRTIs), non-nucleoside reverse transcriptase inhibitors (NNRTIs), and protease inhibitors (PIs). However, HIV remains a significant burden on several countries.^3^ One of the biggest obstacles in the eradication of HIV is the capability of the virus to remain dormant in CD4 + cells during the latent phase. As antiretroviral therapy (ART) targets different stages of viral replication, those within the latent CD4 + T cells can escape the action of ARTs.^5^

 Latency-reversing agents (LRA) were used in conjunction with ART to overcome this. It was introduced as the “shock and kill” strategy.^6,7^ The principle of this was to activate the latent viruses using the LRAs in the “shock” phase, allowing the host defense mechanisms to “kill” the infected cells. Several classes of LRAs have been developed, including PKC agonists, CCR5 antagonists, PI3K/AKT pathway inhibitors, and SMAC analogs.^7^ However, studies with these did not report a significant decline in the latent cells. In addition to this, the LRAs were not specific to HIV, resulting in damage to other healthy host immune cells.^7^

**Figure 1:**
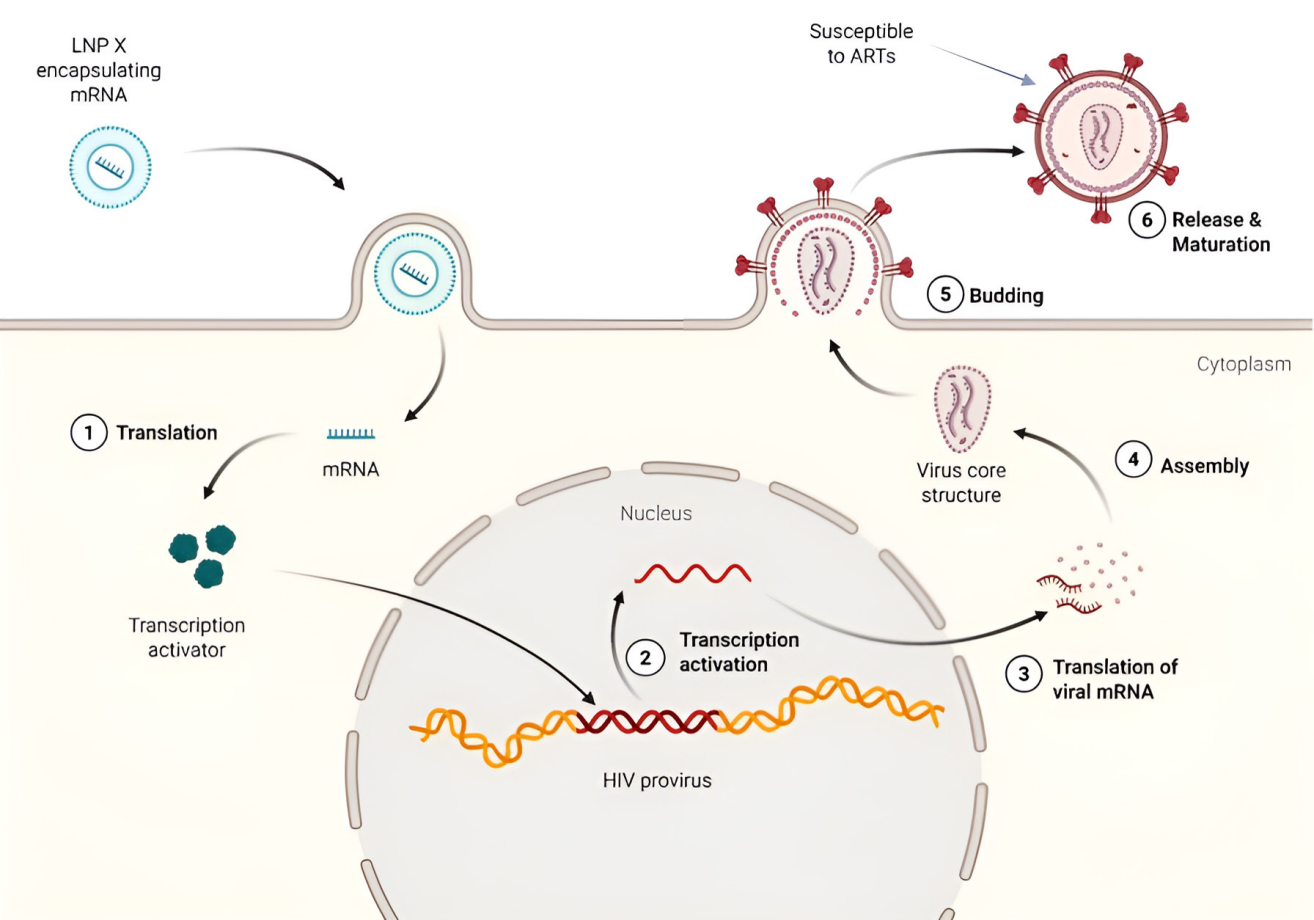
Transcription activation of the HIV provirus by LNP X

 It was reported that although LRAs were able to initiate the process of HIV transcription, it was unable to overcome the subsequent blocks in transcription elongation, completion, and splicing. To overcome these problems, the focus of research shifted to HIV-specific nucleic acid-based LRAs. Among this, lipid nanoparticle (LNP) encapsulating mRNA encoding Trans-activator of Transcription (Tat) was observed to enhance the process of transcription. Another was HIV LTR-targeted CRISPR activation (CRISPRa), which provided highly specific activation of transcription.^5^ However, due to the lack of a delivery system to the T cells, these LRAs have not progressed to further stages of clinical trials. A new study done in 2025, modified FDA-approved LRA, patisiran, to create a new formulation, LNP X.^8^ This new LRA, based on the combination of ionizable, synthetic amino lipid (SM-102) and phytosterol (β-sitosterol), was observed to have a superior potency, with transfection efficiencies being > 75%.^8^ The cellular uptake of LNP X was observed to be 2.5 times higher than the previous LRAs, with protein expression being 4.1 times higher relative to the LNP X that entered the cells.^8^ In addition to this, this research does not demonstrate any in vitro toxicity associated with treatment with LRA-LNP X. It was noteworthy that this mRNA-based LNP X coding Tat - HIV protein was shown to increase the transcription of the virus in ex vivo CD4 + cells of HIV infected individuals (Figure 1). LNP X also enables the delivery of CRISPRa, allowing modulation of viral and host gene transcription. This is the first time that such a significant result has been observed in the therapy targeting HIV latent cells, and it offers potential for the development of nucleic acid-based T cell therapies.^9,10^

## Conclusion

 There are many anti-retroviral drugs are currently available for the treatment of HIV infected individuals. But these drugs are effective only during replicative stages or on assembled HIV virions in the host cells. These drugs are generally ineffective on the latent HIV genome. For successful HIV treatment, it is particularly crucial to detach the HIV provirus attached to the human genome. Lipid nanoparticle-based mRNA technology with LNP X-HIV Tat-CRISPRa system can deliver the mRNA and induces the viral transcription. After detachment, the subsequent replicative stages of HIV are vulnerable and destroyed by the antiretroviral drugs. LNP X-HIV Tat-CRISPRa system in combination with ART shows a promising scope for better treatment strategy of HIV infected individuals.

## Competing Interests

 The authors declare that there are no conflicts of interest.

## Ethical Approval

 Not applicable.

## References

[1] Boomgarden AC, Upadhyay C (2025). Progress and challenges in HIV-1 vaccine research: a comprehensive overview. Vaccines (Basel).

[2] Zhang P, Singh M, Becker VA, Croft J, Tsybovsky Y, Gopan V (2025). Inclusion of a retroviral protease enhances the immunogenicity of VLP-forming mRNA vaccines against HIV-1 or SARS-CoV-2 in mice. Sci Transl Med.

[3] Joint United Nations Programme on HIV/AIDS (UNAIDS). UNAIDS Data 2024. UNAIDS; 2024. Available from: https://www.unaids.org/en/resources/documents/2024/2024_unaids_data. Accessed July 28, 2025.

[4] Swinkels HM, Nguyen AD, Gulick PG. HIV and AIDS. In: StatPearls [Internet]. Treasure Island, FL: StatPearls Publishing; 2025. Available from: https://www.ncbi.nlm.nih.gov/books/NBK534860/. Updated July 27, 2024. Accessed July 28, 2025.

[5] Ghosh AK (2023). Four decades of continuing innovations in the development of antiretroviral therapy for HIV/AIDS: progress to date and future challenges. Glob Health Med.

[6] Fisher BM, Cevaal PM, Roche M, Lewin SR (2025). HIV Tat as a latency reversing agent: turning the tables on viral persistence. Front Immunol.

[7] Zerbato JM, Purves HV, Lewin SR, Rasmussen TA (2019). Between a shock and a hard place: challenges and developments in HIV latency reversal. CurrOpinVirol.

[8] Cevaal PM, Kan S, Fisher BM, Moso MA, Tan A, Liu H (2025). Efficient mRNA delivery to resting T cells to reverse HIV latency. Nat Commun.

[9] Zubair A, Ahmad H, Arif MM, Ali M (2025). mRNA vaccines against HIV: Hopes and challenges. HIV Med.

[10] Hussein M, Molina MA, Berkhout B, Herrera-Carrillo E (2023). A CRISPR-Cas cure for HIV/AIDS. Int J Mol Sci.

